# Utility of the Charlson Comorbidity Index in the Preoperative Evaluation of Patients Undergoing Cardiac Surgery

**DOI:** 10.21470/1678-9741-2025-0151

**Published:** 2026-01-29

**Authors:** Daniel Manzur-Sandoval, Monserrat Echeverria-Ortuño, Rodrigo Gopar-Nieto, Gustavo Rojas-Velasco

**Affiliations:** 1 Cardiovascular Critical Care Unit, Instituto Nacional de Cardiología Ignacio Chávez, Mexico City, Mexico; 2 Coronary Care Unit, Instituto Nacional de Cardiología Ignacio Chávez, Mexico City, Mexico

**Keywords:** Comorbidities, Cardiac Surgical Procedures, Coronary Artery Bypass, Postoperative Period, Age Factors, Risk Factors.

## Abstract

**Introduction:**

The Charlson Comorbidity Index (CCI) is used for assessing comorbidities and estimating risk of adverse outcomes in surgical patients. In cardiac surgery, the burden of comorbidities can significantly influence incidence of postoperative complications and mortality. This study evaluates the utility of CCI in predicting perioperative complications in patients undergoing cardiac surgery.

**Methods:**

Observational cross-sectional study with retrospective data including 483 adult patients who underwent cardiac surgery with cardiopulmonary bypass at the Instituto Nacional de Cardiología Ignacio Chávez from June 2022 to December 2023. Patients were grouped by preoperative CCI: mild (0 - 1), moderate (2), and severe (≥ 3). Statistical analyses (chi-square, Mann-Whitney U, logistic regression) assessed the association between CCI and postoperative complications, adjusting for age and sex.

**Results:**

Patients with severe comorbidity had higher rates of postoperative complications, including delirium (27.3% vs. 9.4%, P = 0.00), stroke (P = 0.03), transfusion (69.7% vs. 47.2%, P = 0.04), and renal replacement therapy (18.2% vs. 5.3%, P = 0.02). Median Sequential Organ Failure Assessment scores at 24 hours were significantly higher (P = 0.00). Logistic regression adjusted for age, sex, and coronary artery bypass grafting identified delirium (odds ratio [OR]: 3.13), nosocomial pneumonia (OR: 3.10), acute kidney injury (OR: 2.28), and renal replacement therapy (OR: 4.10) as independent predictors of severe comorbidity.

**Conclusions:**

The CCI is a valuable tool for predicting postoperative complications in patients undergoing cardiac surgery. Early identification of comorbidities is essential for perioperative planning and optimizing clinical outcomes. Integrating the CCI into routine clinical practice is recommended to enhance patient management.

## INTRODUCTION

**Table:** 

Abbreviations, Acronyms & Symbols				
AIDS	= Acquired immunodeficiency syndrome		HIV	= Human immunodeficiency virus
CABG	= Coronary artery bypass grafting		ICU	= Intensive care unit
CCI	= Charlson Comorbidity Index		IQR	= Interquartile range
CI	= Confidence interval		NYHA	= New York Heart Association
CKD	= Chronic kidney disease		OR	= Odds ratio
COPD	= Chronic obstructive pulmonary disease		SOFA	= Sequential Organ Failure Assessment
EuroSCORE	= European System for Cardiac Operative Risk Evaluation		STS	= Society of Thoracic Surgeons

**Fig. 1:**
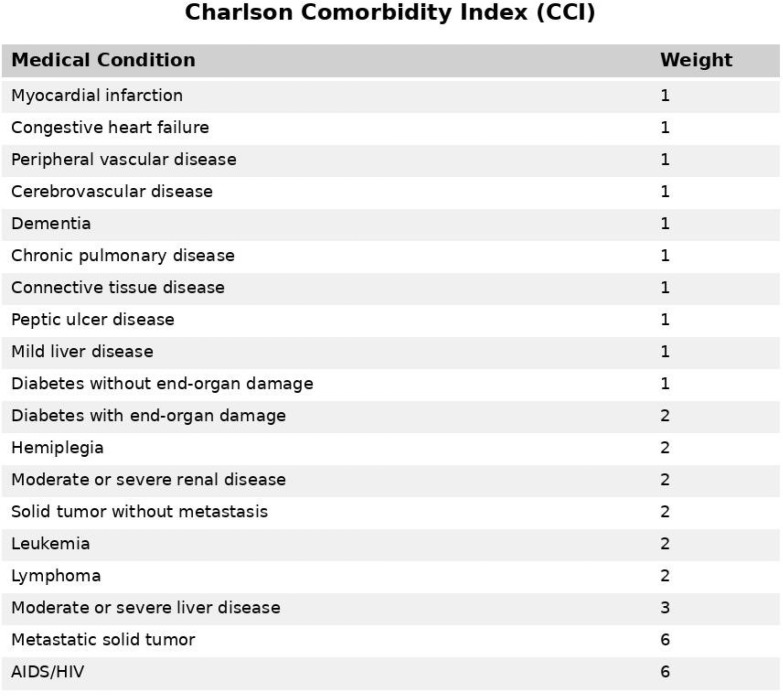
List of medical conditions included in the Charlson Comorbidity Index (CCI), along with their respective weights. AIDS=acquired immunodeficiency syndrome; HIV=human immunodeficiency virus.

The Charlson Comorbidity Index (CCI), developed in 1987 by Mary Charlson et al., has become a fundamental tool for assessing comorbidities in internal medicine. It classifies 17 medical conditions, each assigned a weighted score based on its severity and association with mortality (Figure 1). These conditions range from cardiovascular diseases to malignancies, and the cumulative score is used to predict clinical outcomes and inform therapeutic decision-making^[1]^. The significance of the CCI lies in its ability to provide a comprehensive assessment of a patient’s overall health status, which is particularly relevant in surgical settings, where comorbidities can substantially impact outcomes.

### Importance

The CCI has been validated as a predictor of mortality in diverse patient populations and has become an indispensable tool for risk stratification. Multiple studies show that higher CCI scores are strongly associated with increased postoperative morbidity and long term mortality^[2,3]^. This finding is especially relevant in cardiac surgery, where patients frequently present with several coexisting conditions that complicate perioperative care. Incorporating CCI into routine practice enables clinicians to identify high risk patients who may benefit from intensified monitoring and individualized therapeutic strategies. By accounting for the burden of comorbidities, clinicians can tailor interventions to maximize effectiveness while minimizing adverse effects. Moreover, using CCI at the bedside helps anticipate complications and implement timely preventive measures, thereby improving outcomes^[4,5]^.

Non cardiovascular comorbidities - including diabetes mellitus, peripheral vascular disease, chronic obstructive pulmonary disease (COPD), and chronic kidney disease (CKD) - have a pronounced impact on both short and long term results after cardiac surgery, particularly coronary artery bypass grafting (CABG). In a large contemporary cohort, diabetes was present in nearly 30% of CABG recipients, peripheral vascular disease in 16%, COPD in 18.6%, and renal dysfunction in 27.5%^[6]^. These conditions correlate with advanced age, higher rates of postoperative complications, and reduced access to post discharge interventions such as cardiac rehabilitation, all of which contributing to poorer prognoses. Quantifying this comorbidity burden with the CCI provides a more comprehensive risk profile than traditional cardiovascular risk scores alone.

### Objective

The primary objective of this study is to analyze the utility of CCI in the preoperative setting of cardiac surgery. The study aims to evaluate how the CCI score correlates with postoperative complication rates, mortality, and length of hospital stay. Through an analytical approach, it seeks to determine the predictive capacity of CCI for adverse outcomes in patients undergoing cardiac surgical procedures. Additionally, the study aims to establish recommendations regarding the use of CCI in clinical practice to improve decision-making and perioperative management of at-risk patients. This research will contribute not only to the existing literature on comorbidity assessment in cardiac surgery but also to providing healthcare professionals with practical tools to optimize patient care, ensuring a more comprehensive and safer surgical approach. Given the increasing complexity of patient health profiles, CCI remains a cornerstone in the evaluation and management of comorbidities in the surgical setting.

## METHODS

The study was an analytical observational investigation with retrospective data collection and a cross-sectional design. An open-label approach was employed, with evaluators not blinded to patient information. The sample included adult patients (≥ 18 years) of any sex who underwent cardiac surgery with cardiopulmonary bypass at the Instituto Nacional de Cardiología Ignacio Chávez. A non-probabilistic sampling method was applied, enrolling a total of 483 patients operated on between June 1^st^, 2022, and December 31^st^, 2023. Inclusion criteria were all adult patients undergoing cardiac surgery with cardiopulmonary bypass, without gender restriction. These criteria and sample size were selected to comprehensively represent the target population undergoing this specific surgical intervention during the study period. Patients who died intraoperatively or within the first 12 hours of intensive care unit (ICU) admission were excluded, as our ICU database is designed to capture hemodynamic parameters at 0, six, 12, and 24 hours. Early mortality is often due to unpredictable surgical complications (*e.g.*, uncontrolled bleeding) unrelated to preoperative risk, and including such cases could bias the assessment of preoperative predictors. This exclusion ensured a more homogeneous cohort for risk analysis. Patients with incomplete medical records were also excluded from the study. Data were collected through a detailed review of electronic and physical medical records, as well as electronic imaging studies, to gather demographic, clinical, and surgical information, along with patient outcomes.

In the "other surgeries" group, which individually represented a small proportion of patients, the most frequent procedures included triple valve replacement, aortic surgery (other than Bentall-De Bono), tricuspid valve replacement, congenital heart disease surgery, heart transplantation, pulmonary thromboendarterectomy, endoventricular remodeling, and post-infarction ventricular septal defect closure.

Patients were classified according to their preoperative CCI into three groups consistent with prior studies^[7,8]^:

• Grupo 1: 0 - 1 point, indicating mild comorbidity;
• Group 2: 2 points, considered moderate comorbidity;
• Group 3: ≥ 3 points, indicating severe comorbidity.

For statistical analysis, the normality of continuous variables was assessed using the Shapiro-Wilk test. Parametric variables are presented as mean ± standard deviation, whereas non-parametric variables are reported as median and interquartile range (IQR). Comparisons of continuous variables were performed using the Mann-Whitney U test. Categorical variables were summarized using frequencies and percentages, and comparisons were made using the chi-square test or Fisher’s exact test, depending on expected cell counts. A logistic regression model adjusted for age, sex, and CABG surgery was developed to identify predictors of adverse events. Statistical significance was set at *P* < 0.05 for all analyses, which were conducted using STATA version 14.

### Ethics Approval and Consent to Participate

Local institutional research and ethics committees waived the requirement for approval for this study. Informed consent was obtained from all participants prior to inclusion.

### Consent for Publication

Written informed consent for the publication of patient information and images was obtained either from the patient or a legally authorized representative.

## RESULTS

### Baseline Characteristics

A total of 483 patients were classified by comorbidity severity into mild (n = 395), moderate (n = 55), and severe (n = 33) groups. Arterial hypertension prevalence was higher in moderate (58.2%) and severe (63.6%) groups (*P* = 0.00). Diabetes mellitus was more frequent in moderate (41.8%) and severe (69.7%) groups (*P* = 0.00), with target organ damage present in 66.7% of severe cases (*P* = 0.00). CKD was observed in 2.5% of mild and 57.6% of severe patients (*P* = 0.00), with moderate-to-severe stages in 60.6% of the severe group (*P* = 0.00). Previous myocardial infarction affected 36.4% of severe *vs.* 7.6% of mild patients (*P* = 0.00). Heart failure and cerebrovascular disease were also significantly more prevalent in the severe group (49.1% *vs.* 22.3% and 39.4% *vs.* 5.3%, respectively; *P* = 0.00). Median age was 57 years (IQR 45 - 65), with 76.6% aged 41 - 70 years (Table 1).

**Table 1:** Baseline characteristics.

Variable		Total n = 483	Group 1 n = 395	Group 2 n = 55	Group 3 n = 33	*P*-value
Women, n (%)		207 (42.9)	173 (43.8)	21 (38.2)	13 (39.4)	0.67
Men, n (%)		276 (57.1)	222 (56.2)	34 (61.8)	20 (60.6)	
Hypertension, n (%)		213 (44.1)	160 (40.5)	32 (58.2)	21 (63.6)	< 0.001
Diabetes, n (%)		120 (24.8)	74 (18.7)	23 (41.8)	23 (69.7)	< 0.001
Diabetes with target organ damage, n (%)		31 (6.4)	0	9 (16.4)	22 (66.7)	0.00
Moderate to severe chronic kidney disease, n (%)		36 (7.4)	5 (1.3)	11 (20)	20 (60.6)	0.00
Chronic obstructive pulmonary disease, n (%)		18 (3.7)	5 (1.3)	7 (12.7)	6 (18.2)	< 0.001
Hypothyroidism, n (%)		71 (14.7)	58 (14.7)	7 (12.7)	6 (18.2)	0.78
Previous myocardial infarction, n (%)		54 (11.2)	30 (7.6)	12 (21.8)	12 (36.4)	< 0.001
Heart failure, n (%)		128 (26.5)	88 (22.3)	27 (49.1)	13 (39.4)	< 0.001
Atrial fibrillation, n (%)		101 (20.9)	80 (20.2)	11 (20)	10 (30.3)	0.38
Stroke, n (%)		47 (9.7)	21 (5.3)	13 (23.6)	13 (39.4)	< 0.001
Hemiplegia, n (%)		20 (4.1)	0	8 (14.5)	12 (36.4)	< 0.001
Previous cardiac surgery, n (%)		52 (10.8)	41 (10.4)	8 (14.5)	3 (9.1)	0.61
Peripheral vascular disease, n (%)		38 (7.9)	20 (5.1)	11 (20)	7 (21.2)	< 0.001
Connective tissue disease, n (%)		28 (5.8)	16 (4)	7 (12.7)	5 (15.1)	< 0.001
Peptic ulcer disease, n (%)		29 (6)	15 (3.8)	7 (12.7)	7 (21.2)	< 0.001
Moderate to severe liver disease, n (%)		5 (1)	0	2 (3.6)	3 (9.1)	< 0.001
Mild liver disease, n (%)		6 (1.2)	1 (0.2)	1 (1.8)	4 (12.1)	< 0.001
Solid tumor, n (%)		13 (2.7)	5 (1.3)	5 (9.1)	3 (9.1)	< 0.001
NYHA functional class, n (%)	I	65 (13.5)	54 (13.7)	5 (9.1)	6 (18.2)	0.04
	II	296 (61.3)	248 (62.8)	27 (49.1)	21 (63.6)	
	III	109 (22.6)	85 (21.5)	20 (36.4)	4 (12.1)	
	IV	13 (2.7)	8 (2)	3 (5.4)	2 (6.1)	
Age (years), median (IQR)		57 (45 - 65)	56 (44 - 65)	60 (51-67)	62 (54 - 66)	< 0.001
Weight (kg), median (IQR)		68.5 (60 - 78)	68.8 (60 - 78)	66 (59-77)	73 (60 - 80)	0.49
Height (m), median (IQR)		1.62 (1.55 - 1.7)	1.62 (1.55 - 1.7)	1.62 (1.55-1.7)	1.63 (1.57 - 1.68)	0.95
Body mass index (kg/m^2^), median (IQR)		26.1 (23.4 - 28.9)	26 (23.3 - 28.8)	26.4 (23.6-29.3)	26.4 (23.6 - 29)	0.72

IQR=interquartile range; NYHA=New York Heart Association

### Surgical Characteristics

Aortic valve replacement was performed in 29.4% of patients, with no significant differences between groups (*P* = 0.12). CABG was performed in 15.5% of patients and was more frequent in the moderate (23.6%) and severe (33.3%) comorbidity groups compared to the mild group (12.9%) (*P* < 0.001). Mitral valve replacement was performed in 9.5% of patients, with no significant differences between groups (*P* = 0.74). The European System for Cardiac Operative Risk Evaluation (EuroSCORE) had a median value of 1.8 (IQR: 1-3.4), with significant increases observed in the moderate (2.2) and severe (2.5) groups (*P* < 0.001). The median cardiopulmonary bypass time was 144 minutes (IQR: 112 - 186), with no significant differences between groups (*P* = 0.50). Similarly, the median aortic cross-clamping time was 100 minutes (IQR: 76 - 125), also showing no significant differences (*P* = 0.26) (Table 2).

**Table 2:** Surgical characteristics.

Variable	Total n = 483	Group 1 n = 395	Group 2 n = 55	Group 3 n = 33	*P*-value
Aortic valve replacement, n (%)	142 (29.4)	124 (31.4)	11 (20)	7 (21.2)	0.12
Coronary artery bypass grafting, n (%)	75 (15.5)	51 (12.9)	13 (23.6)	11 (33.3)	< 0.001
Mitral valve replacement, n (%)	46 (9.5)	38 (9.6)	6 (10.9)	2 (6.1)	0.74
Mitral valve replacement + tricuspid valve replacement, n (%)	26 (5.4)	19 (4.8)	4 (7.3)	3 (9.1)	0.46
Aortic valve replacement + mitral valve replacement, n (%)	31 (6.4)	27 (6.8)	3 (5.4)	1 (3)	0.87
CABG + aortic valve replacement, n (%)	20 (4.1)	17 (4.3)	2 (3.6)	1 (3)	1
Bentall-De Bono procedure, n (%)	25 (5.2)	23 (5.8)	2 (3.6)	0	0.39
Other surgeries, n (%)	121 (25)	97 (24.6)	15 (27.3)	9 (27.3)	0.82
Cardiopulmonary bypass time (minutes), median (IQR)	144 (112 - 186)	143 (113 - 187)	149 (105 - 208)	140 (97 - 175)	0.50
Aortic cross-clamping time (minutes), median (IQR)	100 (76 - 125)	101 (77 - 124)	98 (75 - 134)	87 (65 - 113)	0.26
EuroSCORE (%), median (IQR)	1.8 (1 - 3.4)	1.6 (0.9 - 3.2)	2.2 (1.5 - 4.9)	2.5 (1.5 - 6.9)	< 0.001
STS score (%), median (IQR)	0.9 (0.5 - 1.7)	0.8 (0 - 1)	1.1 (0.9 - 1.4)	1.3 (1 - 1.6)	< 0.001

CABG=coronary artery bypass grafting; EuroSCORE=European System for Cardiac Operative Risk Evaluation; IQR=interquartile range; STS=Society of Thoracic Surgeons

### Postoperative Complications

A higher incidence of postoperative complications was observed in the severe comorbidity group. Delirium occurred in 27.3% of patients with severe comorbidities compared to 9.4% in the mild group (*P* = 0.00). Stroke was more frequent in the moderate (9.1%) and severe (6.1%) groups than in the mild group (2.8%) (*P* = 0.03). Hospital-acquired pneumonia affected 12.7% of patients in the moderate group *vs.* 8.9% in the mild group (*P* = 0.01). Transfusion rates were significantly higher in the severe group (69.7%) compared to the mild group (47.2%) (*P* = 0.04). Renal replacement therapy was more common in the severe group (18.2%) than in the mild group (5.3%) (*P* = 0.02). The median Sequential Organ Failure Assessment score at 24 hours was higher in the severe group (7 [IQR 4-8]) compared to the mild group (5 [IQR 3-7]) (*P* = 0.00), with a trend toward higher scores at 72 hours (*P* = 0.05). Although acute kidney injury was more frequent in the severe group (48.5%) than in the mild group (29.4%), this difference did not reach statistical significance (*P* = 0.06). No significant differences were found in mediastinal hemorrhage, low cardiac output syndrome, vasoplegic syndrome, hypovolemia, mediastinitis, hepatic dysfunction, postoperative atrial fibrillation, in-hospital mortality, ICU stay, mechanical ventilation duration, or total hospital length of stay (Table 3).

**Table 3:** Outcomes.

Variable	Total	Group 1	Group 2	Group 3	*P*-value
	n = 483	n = 395	n = 55	n = 33	
Mediastinal hemorrhage, n (%)	56 (11.6)	46 (11.6)	5 (9.1)	5 (15.1)	0.68
Low cardiac output syndrome, n (%)	59 (12.2)	47 (11.9)	7 (12.7)	5 (15.1)	0.85
Vasoplegic syndrome, n (%)	33 (6.8)	27 (6.8)	2 (3.6)	4 (12.1)	0.35
Hypovolemia, n (%)	184 (38.1)	151 (38.2)	18 (32.7)	15 (45.4)	0.48
Delirium, n (%)	57 (11.8)	37 (9.4)	11 (20)	9 (27.3)	< 0.001
Stroke, n (%)	18 (3.7)	11 (2.8)	5 (9.1)	2 (6.1)	0.03
In-hospital pneumonia, n (%)	50 (10.4)	35 (8.9)	7 (12.7)	8 (2.42)	0.01
Mediastinitis, n (%)	21 (4.4)	15 (3.8)	4 (7.3)	2 (6.1)	0.32
Transfusion, n (%)	235 (48.8)	186 (47.2)	26 (47.3)	23 (69.7)	0.04
Acute kidney injury, n (%)	147 (30.5)	116 (29.4)	15 (27.3)	16 (48.5)	0.06
Renal replacement therapy, n (%)	29 (6)	21 (5.3)	2 (3.6)	6 (18.2)	0.02
Hepatic dysfunction, n (%)	61 (12.7)	56 (14.2)	4 (7.3)	1 (3)	0.08
Postoperative atrial fibrillation, n (%)	76 (15.8)	60 (15.2)	10 (18.2)	6 (18.2)	0.79
In-hospital mortality, n (%)	28 (5.8)	22 (5.6)	3 (5.4)	3 (9.1)	0.63
Postoperative ICU stay (days), median (IQR)	3 (2 - 4)	3 (2 - 4)	2 (2 - 4)	3 (2 - 5)	0.19
Days on mechanical ventilation, median (IQR)	1 (1 - 1)	1 (1 - 1)	1 (1 - 1)	1 (1 - 2)	0.55
Hospital length of stay (days), median (IQR)	10 (7 - 17)	9 (7 - 16)	12 (7 - 21)	11 (7 - 21)	0.09
SOFA score 24 hours, median (IQR)	5 (3 - 7)	5 (3 - 7)	4 (3 - 7)	7 (4 - 8)	< 0.001
SOFA score 72 hours, median (IQR)	3 (2 - 5)	3 (2 - 5)	4 (2 - 6)	4 (2 - 7)	0.05

ICU=intensive care unit; IQR=interquartile range; SOFA=Sequential Organ Failure Assessment

### Logistic Regression Analysis

Logistic regression analyses were conducted to identify factors associated with severe comorbidity, adjusting for age, sex, and CABG surgery. Due to the statistically significant difference in CABG distribution across comorbidity groups (33.3% in the severe group *vs.* 12.9% in the mild group; *P* < 0.001), CABG was included as a covariate in the multivariable logistic regression model to control for potential confounding. Postoperative delirium (odds ratio [OR]: 3.13; 95% confidence interval [CI]: 1.37 - 7.13; *P* = 0.001) and nosocomial pneumonia (OR: 3.10; 95% CI: 1.31 - 7.30; *P* = 0.01) were significantly associated with an increased risk of severe comorbidity. Acute kidney injury also showed a significant association (OR: 2.28; 95% CI: 1.12 - 4.65; *P* = 0.02). Renal replacement therapy was associated with the highest odds ratio (OR: 4.10; 95% CI: 1.54 - 10.93; *P* < 0.001), indicating a more than fourfold increased risk (Table 4).

**Table 4:** Logistic regression model adjusted for age, sex, and CABG surgery.

Variable	OR	95% CI	*P*-value
Delirium	3.13	1.37 - 7.13	< 0.001
In-hospital pneumonia	3.10	1.31 - 7.30	0.01
Acute kidney injury	2.28	1.12 - 4.65	0.02
Renal replacement therapy	4.10	1.54 - 10.93	< 0.001

CABG=coronary artery bypass grafting; CI=confidence interval; OR=odds ratio

## DISCUSSION

This study highlights the importance of CCI as a key tool in the preoperative assessment of patients undergoing cardiac surgery, particularly when compared with more commonly used risk scores such as EuroSCORE and the Society of Thoracic Surgeons (STS) score. Our results suggest that the CCI could have a significant impact on predicting postoperative complications by incorporating a broad spectrum of comorbidities, not limited to cardiac conditions alone. The high prevalence of diseases such as hypertension and diabetes in our cohort underscores the need to integrate tools that assess the risk associated with these comorbidities, which are crucial for effective preoperative management.

Regarding the performance of conventional risk scores, previous studies have shown that both EuroSCORE and STS score have limited discriminatory power in certain populations. Nilsson et al.^[9]^ reported that EuroSCORE is among the scores with the greatest discriminatory ability in cardiac surgery, closely followed by the Cleveland Clinic score and the Magovern score. Despite their widespread acceptance, these tools may not be sufficient to optimally predict postoperative outcomes in patients with multiple comorbidities. The CCI, by accounting for comorbidities beyond cardiovascular diseases, could offer a more robust prediction, particularly in patients with multiple chronic conditions. It is important to emphasize that, although EuroSCORE and STS score are more specific to cardiovascular procedures, their performance is limited in patients with significant non-cardiac comorbidities, as reflected in our study’s findings. For example, the incidence of severe complications such as delirium and acute kidney injury was higher in patients with elevated CCI scores - outcomes that are not always adequately captured by traditional scores.

The application of CCI could potentially reduce the rates of these severe complications, which substantially increase the costs associated with postoperative care. Moreover, improved prediction of complications would allow for optimized resource allocation and more efficient tailoring of interventions, potentially enhancing survival and reducing long-term healthcare costs in patients with multiple chronic conditions^[10-14]^. Our findings suggest that CCI may offer an advantage in clinical settings where patients present with multiple comorbidities. By integrating a broader evaluation of overall patient health status, CCI could enable more precise personalization of perioperative care, tailoring management strategies according to the specific risks each patient faces, including those from conditions beyond the cardiovascular system.

### Limitations

This study has several inherent limitations. Firstly, its retrospective design introduces potential selection and recording biases. Additionally, the absence of follow-up data limits the ability to evaluate the temporal progression of patients. While associations between variables can be identified in this type of study, causal relationships cannot be established. Furthermore, as a single-center study, the findings may not be generalizable to other populations or settings.

## CONCLUSION

The CCI has proven to be a valuable tool for predicting complications in patients undergoing cardiac surgery. Early identification of comorbidities can guide clinical decision-making and improve patient outcomes. It is essential for healthcare professionals to incorporate comorbidity assessment into the surgical care process, as this not only enhances the quality of care but also positively influences survival and quality of life in this patient population.

## Data Availability

The authors declare that the data supporting the findings of this study are available within the article.
